# Pharmacokinetic/pharmacodynamic analysis to characterize the effect of long-term GlyT1 inhibitor iclepertin exposure on hemoglobin levels

**DOI:** 10.3389/fphar.2026.1770980

**Published:** 2026-07-10

**Authors:** Nina Hanke, Samuel P. Callisto, Peter Nagy, Michael Desch, Mahmoud Tareq Abdelwahab

**Affiliations:** 1 Global Clinical Pharmacology and Non-clinical Safety Science, Boehringer Ingelheim Pharma GmbH & Co. KG, Ingelheim, Germany; 2 Metrum Research Group, Boston, MA, United States; 3 Global Patient Safety and Pharmacovigilance, Boehringer Ingelheim International GmbH, Ingelheim, Germany

**Keywords:** dose optimization, exposure-safety analysis, glycine transporter-1 (GlyT1), hemoglobin, iclepertin, model-informed drug development (MIDD), pharmacokinetic-pharmacodynamic (PKPD) modeling, safety

## Abstract

**Background:**

This analysis was conducted to assess the risk of anemia in schizophrenia patients during long-term treatment with the glycine transporter-1 (GlyT1) inhibitor iclepertin.

**Methods:**

A population pharmacokinetic-pharmacodynamic (popPKPD) analysis to characterize the impact of iclepertin exposure on hemoglobin levels was performed using a sequential nonlinear mixed effects modeling approach. The effects of patient characteristics were investigated in a covariate analysis to identify vulnerable patient subgroups, and population simulations were conducted to evaluate different treatment scenarios.

**Results:**

Simulations predicted a new, decreased hemoglobin steady state under chronic iclepertin treatment, reached after approximately 120 days. For a typical patient, the intended therapeutic dose of 10 mg iclepertin daily led to a 2% decrease of hemoglobin levels. In a potential extreme scenario of iclepertin exposure fivefold higher than the average exposure following a 10 mg dose (e.g., due to co-administration of a strong CYP3A4 inhibitor), a 7.6% decrease of hemoglobin levels was found. In both scenarios more than 97.5% of the virtual patients stayed above the drug discontinuation safety threshold of 100 g/L hemoglobin (Phase III trial drug discontinuation threshold defined by the patient safety team). Sex, race, age, body mass index and alanine transaminase levels were found to correlate with changes in hemoglobin levels. No correlation with kidney function could be identified. None of the investigated covariate effects were strong enough to raise any safety concerns during chronic treatment with 10 mg iclepertin daily.

**Conclusion:**

This work provides a generalizable modeling and simulation framework to assess the anemia risk in patients and vulnerable patient subgroups during chronic iclepertin treatment. The results of this analysis suggest that iclepertin drug effects on patient hemoglobin levels are small, reversible, and of limited significance, even under long-term treatment. This is the first model of the relationship between iclepertin exposure and hemoglobin levels, and, to our best knowledge, the first model to characterize a drug effect on hemoglobin levels over time that has been validated with clinical data that extend beyond the erythrocyte life span of ∼126 days.

## 1 Introduction

Positive and negative symptoms are typically emphasized in the diagnosis of schizophrenia, but cognitive impairment is an important dimension of the illness which constitutes a high burden for the affected patients, e.g., due to school dropout or loss of employment. Currently, there are no pharmacological options for the treatment of cognitive impairment associated with schizophrenia (Rosenbrock et al., 2023). Iclepertin (BI 425809) is a selective glycine transporter-1 (GlyT1) inhibitor that recently has been investigated in Phase III trials for the treatment of cognitive impairment associated with schizophrenia (CIAS) in adults at a daily dose of 10 mg (CONNEX-1: NCT04846868, CONNEX-2: NCT04846881, CONNEX-3: NCT04860830). Findings from the Phase III trials confirmed the favorable safety profile of iclepertin observed during Phase I and II development, but the Phase III efficacy endpoints were not met (Moschetti et al., 2018a; Moschetti et al., 2018b; Fleischhacker et al., 2021; Keefe et al., 2025; Nagy et al., 2026).

While inhibition of GlyT1 in the brain was foreseen to improve cognition in CIAS patients by increasing the concentration of the NMDA receptor co-activator glycine in the synaptic cleft, inhibition of GlyT1 in the cell membrane of erythrocyte precursors could possibly interfere with glycine uptake for heme biosynthesis and consequently affect the production of hemoglobin. Reversible time- and dose-dependent decreases of hemoglobin have been observed in clinical studies with a different GlyT1 inhibitor named bitopertin and seem to be a class effect (Umbricht et al., 2014; Hirayasu et al., 2016).

Population pharmacokinetic-pharmacodynamic (popPKPD) modeling has been used previously to describe the relationship between drug exposure and its effect on hemoglobin. Hamrén et al. published a mechanism-based PKPD model to characterize the impact of tesaglitazar exposure on fasting plasma glucose, hemoglobin and glycosylated hemoglobin in patients with type 2 diabetes mellitus (Hamrén et al., 2008). Schaedeli Stark et al. presented a semi-physiologic PKPD model to characterize the effect of bitopertin on hemoglobin, taking the feedback on erythrocyte proliferation into account (Schaedeli-Stark et al., 2012). Recently, Rognås et al. followed up on this model, extending it to include descriptions of the corresponding reticulocyte counts and immature reticulocyte fractions using data from the same clinical trial (Rognås et al., 2025).

The presented popPKPD model was developed to assess the risk of anemia in patients during long term iclepertin treatment. Specific objectives of our analysis were to simulate the hemoglobin concentration-time course under chronic iclepertin treatment (365 days) with the intended therapeutic dose of 10 mg daily, as well as a potential extreme scenario of fivefold higher steady state exposure [e.g., due to co-administration of a strong CYP3A4 inhibitor (Matsson et al., 2025)], and to investigate the effects of patient characteristics to assess if there is a need for hemoglobin monitoring in vulnerable patient subgroups.

## 2 Materials and methods

### 2.1 Data

#### 2.1.1 Clinical data used for model development

First, a popPK model to describe the pharmacokinetics (PK) of iclepertin was developed, using the clinical data of seven Phase I and two Phase II trials (see Supplementary Table S1 for details).

Then, a popPD model of the relationship between iclepertin exposure and hemoglobin levels was built based on the data of these trials, but since the effect of iclepertin on hemoglobin is slow, only the three studies providing long-term treatment information were included for the popPD part. Table 1 summarizes the treatment durations, numbers of subjects per dose group and sampling times. We combined data from healthy individuals, CIAS patients and Alzheimer’s disease patients for model development, assuming that there is no difference in the role of GlyT1 in hemoglobin synthesis between these populations. Together, these trials contain clinical data from 1116 individuals treated with placebo or iclepertin doses ranging from 2 to 50 mg daily with at least one post-dose measurement of hemoglobin concentrations (HGB), red blood cell counts (RBC) and mean corpuscular hemoglobin (MCH). Supplementary Figure S1 shows the individual hemoglobin measurements over time from these three trials.

**TABLE 1:** Clinical data used for popPD model development.

Study type	Dose groups	Treatment duration	Number of subjects (no. per dose group)	Planned HGB, RBC, MCH samples [day]
Clinical model building data				
Proof of mechanism	5 mg, 10 mg, 25 mg, 50 mg daily	14 days (+14 days follow-up)	n = 25, healthy volunteers (6/6/8/5)	−20, 0, 2, 6, 10, 13, +3, +14
Phase II in CIAS	Placebo, 2 mg, 5 mg, 10 mg, 25 mg daily	12 weeks (+4 weeks follow-up)	n = 492, CIAS patients (170/78/81/83/80)	−20, 0, 42, 84, +28
Phase II in AD	Placebo, 2 mg, 5 mg, 10 mg, 25 mg daily	12 weeks (+4 weeks follow-up)	n = 599, AD patients (118/120/118/122/121)	−20, 0, 28, 56, 84, +28
Clinical model validation data				
Phase III CONNEX-1/-2/-3	Placebo, 10 mg daily	26 weeks (+4 weeks follow-up)	n = 1682, CIAS patients (861/821)	−28, 0, 42, 84, 182, +28

AD: Alzheimer’s disease, CIAS: Cognitive impairment associated with schizophrenia, HGB: Hemoglobin, MCH: Mean corpuscular hemoglobin, RBC: Red blood cell count. Drug administration at time = 0, after the pre-dose sample. Placebo groups excluded for model development. Proof of Mechanism: NCT02362516, Phase II, in CIAS: NCT02832037, Phase II, in AD: NCT02788513. CONNEX-1: NCT04846868, CONNEX-2: NCT04846881, CONNEX-3: NCT04860830.

Following the popPD model building, HGB, RBC and MCH measurements of 821 patients treated with 10 mg iclepertin daily for 26 weeks in the CONNEX Phase III trials were used as external validation cohort to verify the generalizability of the popPD model.

### 2.2 Software

The popPKPD analysis was performed using non-linear mixed-effect modeling in NONMEM 7.4.3 (ICON Development Solutions, Ellicott City, MD), and Perl speaks-NONMEM 4.8.1 (PsN).

Data handling, calculations, visualizations, and simulations with mrgsolve 1.5.1 (Baron, 2025) were performed in R version 4.4.1 (R-Core-Team, 2021).

### 2.3 Model building

The population pharmacokinetic (popPK) and pharmacodynamic (PD) models were developed sequentially. First, the PK of the iclepertin tablet formulation at clinically relevant dose levels was characterized using measurements from nine different clinical studies (listed in Supplementary Table S1). The parameter estimates of the popPK model are shown in Supplementary Table S2. Geometric mean steady state exposure predictions for the Phase II in CIAS patient population are presented in Supplementary Table S3. This popPK model was then applied to generate individual steady state PK exposure values as input for PD model development, taking the effects of the identified PK covariates into account, and a semi-mechanistic model to describe the relationship between iclepertin exposure and hemoglobin levels was established.

The first-order conditional estimation method with interaction (FOCE-I) was used for parameter estimation. Model selection was based on model convergence, NONMEM objective function value (OFV), precision of parameter estimates and condition number. A nested model was considered superior to another, if the OFV was reduced by >3.84 units (chi-square test statistic, p<0.05, 1 degree of freedom). In addition, goodness-of-fit plots, individual plots and visual predictive checks (bootstraps with 1000 samples) were assessed.

#### 2.3.1 Structural model

The model published by Schaedeli-Stark et al. was used as a starting base model (Schaedeli-Stark et al., 2012). Bitopertin, like iclepertin, is a selective GlyT1 inhibitor that decreases hemoglobin synthesis (class effect). The bitopertin model was developed on clinical data from a study applying 120 days of treatment (daily dosing, placebo and 3 different dose arms). The bitopertin clinical data also did not answer the question of when the new hemoglobin concentration steady state will be reached under chronic treatment.

Individual area under the iclepertin plasma concentration-time curve at steady state (AUC_τ,ss_) values were used as exposure metric to drive the drug effect on hemoglobin (Hamrén et al., 2008; Schaedeli-Stark et al., 2012; Rognås et al., 2025), predicted with the individual Bayesian PK parameter estimates from the popPK model. The exposure-response relationship between iclepertin AUC_τ,ss_ and hemoglobin was then modeled in the popPKPD model. Iclepertin PK steady state is reached quickly compared to the red blood cell lifespan, the elimination half-life of iclepertin being 33–47 h (Moschetti et al., 2018a) compared to the ∼126-day lifespan of red blood cells. Different assumptions on iclepertin exposure for the time after treatment stopped were tested: AUC_τ,ss_ set to zero 24 h after the last dose, or AUC_τ,ss_ set to zero 48 h after the last dose.

**FIGURE 1:**
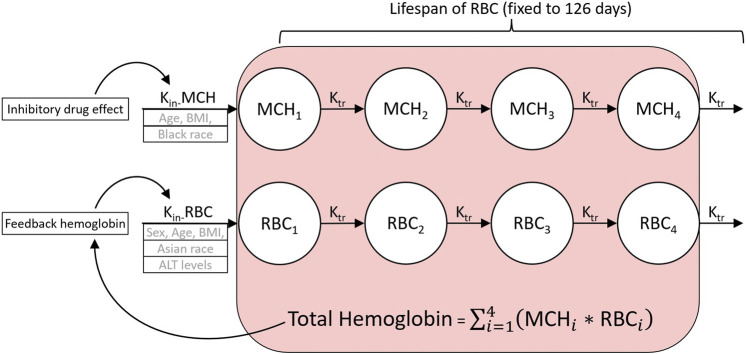
Diagram of the structural model, illustrating the two parallel transit compartment chains to enable incorporation of (i) the inhibitory drug effect on hemoglobin content within the red blood cells (MCH), and (ii) of the homeostatic feedback mechanism of total hemoglobin on red blood cell (RBC) proliferation. ALT: alanine transaminase, BMI: body mass index, K_in__MCH: MCH production rate, K_in__RBC: RBC production rate, K_tr_: transit compartment transfer rate, MCH: mean corpuscular hemoglobin, RBC: red blood cell count.

We aimed to create a model structure with sufficient physiological detail to capture both the effect of iclepertin GlyT1 inhibition on hemoglobin synthesis and the homeostatic feedback of hemoglobin levels on erythrocyte proliferation. To accomplish this, the model was structured as two related transit-compartment chains: one describing hemoglobin content in red blood cells (mean corpuscular hemoglobin, MCH) and one describing the red blood cell count (RBC; Figure 1). For parameter estimation, the model simulations were simultaneously fit to the observed individual MCH and RBC measurements over time (including pre-treatment samples).

Different implementations of the inhibitory drug effect on hemoglobin synthesis were tested (subtractive effect on MCH production rate, proportional effect on MCH production rate, linear inhibition, E_max_ model inhibition). Furthermore, we assessed different implementations of the known physiological feedback mechanism in which changes in hemoglobin levels regulate RBC proliferation to maintain tissue oxygenation homeostasis (absolute change of hemoglobin level as driver, relative change of hemoglobin level as driver, proportional effect on RBC proliferation rate, exponential effect on RBC proliferation rate).

#### 2.3.2 Statistical model

Interindividual variability (IIV) on estimated parameters was tested using exponential error models for between subject random effects. Residual unexplained variability was implemented using additive error models.

#### 2.3.3 Covariate assessment

First, the PK covariates were analyzed and implemented into the popPK model. Using this model to generate individual subject’s Bayes exposure estimates as input for the popPD model development accounts for the individual variability in drug exposure and separates the PK covariate effects from the PD covariate analysis.

The evaluated PD covariates were selected based on mechanistic plausibility, exploratory data analysis and clinical interest, and included sex, race, age, body weight, height, body mass index (BMI), lean body weight, alanine transaminase (ALT), aspartate transaminase (AST), estimated glomerular filtration rate (eGFR), serum creatinine, total serum protein, alcohol consumption history, smoking status, medical diagnosis, and daily dose.

First, graphical analyses of individual IIV values versus the corresponding individual covariate values were conducted for the selected covariates. If there was a trend in a graphical analysis, it was tested if implementation of the covariate on the respective model parameter improved the model fit and reduced the associated IIV.

Stepwise inclusion was performed in the order of statistical significance, avoiding implementation of correlated covariates such as body weight and BMI. Retention in the final model was decided based on the same criteria as applied during base model selection (OFV reduced by >3.84 units, p<0.05, see Section 2.3 “Model building”). Covariates for the final model should be clinically plausible, and meaningful with regard to their effect size compared to the observed inter- and intra-subject variability in the observed data.

**FIGURE 2:**
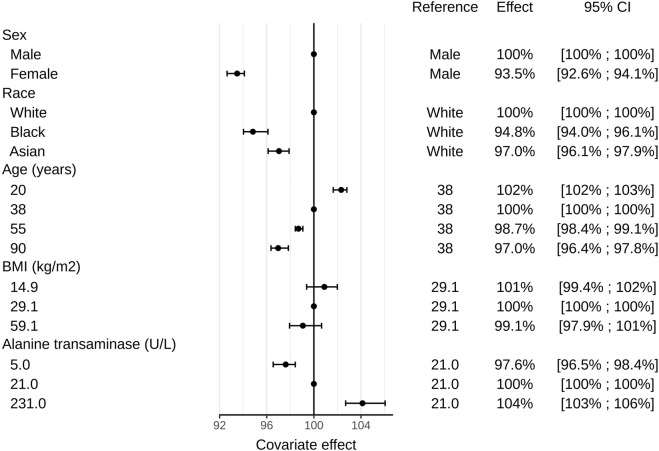
Effects of covariates on hemoglobin concentrations at day 365 of treatment with 10 mg iclepertin daily. Closed circles represent the effect at the median population estimate, error bars represent the 95% confidence interval (median and 95% confidence interval derived from a PsN bootstrap with 1000 samples). The black vertical line represents the Phase II CIAS trial reference patient: male, White, 38 years of age, BMI = 29.1 kg/m^2^, ALT = 21.0 U/L. BMI and ALT were varied to the minimum and maximum values of the Phase II CIAS and Phase II AD patient populations.

The covariate effects on model endpoints of interest were visualized in covariate-effect plots (“forest-like plots”). Only one covariate was varied at a time (univariate evaluation), while the other covariates were set to the median demographic or laboratory values of the Phase II CIAS patient population (“reference patient”). Then, the percent change relative to the reference covariate set was plotted. The forest-like plots were generated from simulations of the endpoints of interest (e.g., pretreatment hemoglobin levels, hemoglobin levels after 365 days of treatment), based on the median population estimates and associated uncertainty obtained from a PsN bootstrap with 1000 samples (Figure 2).

### 2.4 Model-based simulations

**FIGURE 3:**
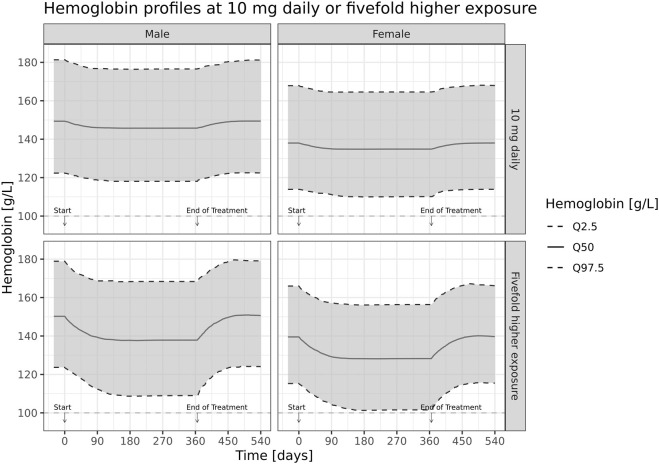
Patient population simulations of hemoglobin during 365 days of treatment with 10 mg iclepertin daily (upper panel) or at a fivefold higher exposure (lower panel), stratified by sex. Black solid lines show the prediction medians, black dashed lines show the 2.5th and 97.5th prediction percentiles. Grey dashed lines indicate the drug discontinuation safety threshold of 100 g/L hemoglobin.

**FIGURE 4:**
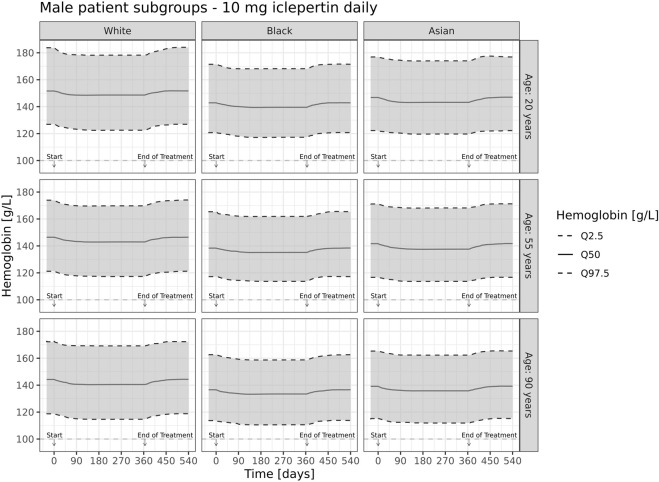
Patient population simulations of hemoglobin during a 1-year treatment of male patients with 10 mg iclepertin daily. Race and age were varied to investigate the risk of impeded hemoglobin synthesis during chronic treatment of different patient subgroups. Black solid lines show the prediction medians, black dashed lines show the 2.5th and 97.5th prediction percentiles. Grey dashed lines indicate the drug discontinuation safety threshold of 100 g/L hemoglobin.

**FIGURE 5:**
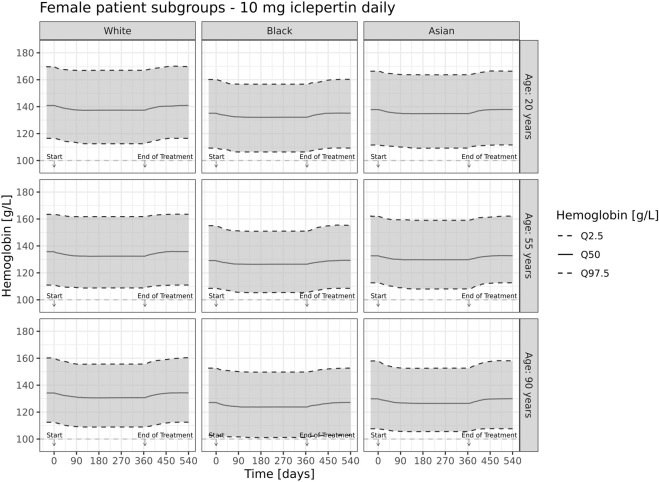
Patient population simulations of hemoglobin during a 1-year treatment of female patients with 10 mg iclepertin daily. Race and age were varied to investigate the risk of impeded hemoglobin synthesis during chronic treatment of different patient subgroups. Black solid lines show the prediction medians, black dashed lines show the 2.5th and 97.5th prediction percentiles. Grey dashed lines indicate the drug discontinuation safety threshold of 100 g/L hemoglobin.

Patient population simulations were performed using the final estimates of the popPKPD model to investigate the risk of impeded hemoglobin synthesis during long-term iclepertin treatment of different patient subgroups. To assess the risk during fivefold elevated exposure we used the Phase III population and re-sampled 1000 male and 1000 female patients to simulate their hemoglobin profiles (Figure 3). To assess the risk during simultaneous variation of sex, race and age, 500 simulations were run per subgroup, to verify that the predicted range of hemoglobin levels did not fall below 100 g/L (Figures 4, 5).

### 2.5 Model validation

The ability of the Phase II popPKPD model to predict future hemoglobin data was investigated using an external validation approach. Individual demographics of the Phase III verum patients were extracted to generate individual AUC_τ,ss_ values using the Phase II popPK model. Subsequently, these AUC_τ,ss_ values were applied as input in the Phase II popPD model to predict their individual hemoglobin concentrations under treatment, considering the actual patient PD covariates. Model predictions were then compared to the Phase III clinical data to confirm the model was able to predict accurately out to 26 weeks in a new set of subjects.

## 3 Results

### 3.1 Final model

Individual AUC_τ,ss_ values, predicted with the individual Bayesian PK parameter estimates from the popPK model, were used as exposure metric to drive the drug effect on hemoglobin (Hamrén et al., 2008; Schaedeli-Stark et al., 2012; Rognås et al., 2025). AUC_τ,ss_ was set to zero 48 h after the last dose, corresponding to approximately one half-life of iclepertin. Since the hemoglobin decrease is dependent on the erythrocyte half-life, keeping the exposure for 24 h longer after the last dosing interval only marginally impacted the AUC_50_ and E_max_ estimates, but resulted in a model with better OFV.

The impact of GlyT1 inhibition on hemoglobin production can only take place in the erythrocyte precursors because soon after entering the vasculature from the bone marrow these precursors lose their ability to synthesize proteins, including hemoglobin. Because of this time window for hemoglobin synthesis (∼7 days) combined with the long lifespan of red blood cells (∼126 days), the hemoglobin concentration in the blood could only slowly decrease during chronic GlyT1 inhibition until a new steady state is reached, requiring all the red blood cells to be replaced with cells that were formed after the beginning of treatment (∼126 days).

The developed model uses two corresponding chains of transit compartments to describe the slow drug effect on hemoglobin: one to represent the hemoglobin load of the red blood cells, and the other to describe the production, lifespan and degradation of the red blood cells themselves (Figure 1). Both chains consist of 4 transit compartments each, with the same first-order transfer rate, derived from the RBC lifespan (K_tr_ = 4/lifespan), shared between all of them. Total hemoglobin is modeled as the product of the mean hemoglobin content per cell and the red blood cell count of the corresponding compartment, summed over all four compartments.

Iclepertin GlyT1 inhibition was modeled as a proportional drug effect (E_max_ model) on the MCH production rate (K_in__MCH): $\frac{dM{CH}_{1}}{dt}=K_{in}\_MCH\times \left(1-\frac{E_{\max}\times AUC}{{AUC}_{50}+AUC}\right)-K_{tr}\times {MCH}_{1}$

where AUC = iclepertin exposure, AUC_50_ = iclepertin exposure leading to half of the maximum drug effect, E_max_ = maximum iclepertin effect, K_tr_ = transit compartment transfer rate, MCH_1_ = first MCH transit compartment. Other mathematical implementations were tested but did not perform as well. A comparison of the respective objective function values is provided in Supplementary Table S5.

The physiological hemoglobin feedback mechanism, where a decrease in hemoglobin leads to increased erythropoietin secretion by the kidneys and thereby stimulation of red blood cell proliferation in the bone marrow, was modeled as a power function on the RBC production rate (K_in__RBC): $\frac{dRBC_{1}}{dt}=K_{in}\_RBC\_0\times {\left(\frac{HB}{{HB}_{-}0}\right)}^{Feedbk}-K_{tr}\times {RBC}_{1}$

where Feedbk = stimulation of RBC production rate as feedback to hemoglobin decrease, HB = hemoglobin time-varying value, HB_0 = baseline hemoglobin, K_in__RBC_0 = baseline RBC production rate, K_tr_ = transit compartment transfer rate, RBC_1_ = first RBC transit compartment.

Regarding the statistical model, the final model includes IIV on the MCH production rate (K_in__MCH), the RBC production rate (K_in__RBC), the AUC_50_ of the inhibitory drug effect (AUC_50_), and the hemoglobin feedback mechanism (Feedbk).

The difference in hemoglobin levels between female and male individuals was implemented through the red blood cell production rate (K_in__RBC). Other identified covariates included age, BMI, race and ALT. The parameter estimates of the final model are listed in Table 2, the NONMEM control stream is provided in the Supplementary Material.

**TABLE 2:** Parameter estimates of the final population pharmacodynamic model.

​	Parameter (unit)	Estimate	RSE%
Fixed effects			
​	Lifespan RBC (days)	126 (fixed)	-
Hemoglobin load	MCH production rate (pg/cell/day)	0.244	0.183
​	E_max_ of inhibitory drug effect ( )	0.552	19.3
​	AUC_50_ of inhibitory drug effect (nmol*h/L)	43500	22.7
Red blood cells	RBC production rate (10^12^/L/day)	0.0351	0.365
​	Feedback on RBC production rate ( )	−1.41	9.46
Covariates K_in__MCH	Effect of age described as $K_{in}\_MCH\times {\left(\frac{AGE}{59}\right)}^{\theta }$	0.0235	20.3
​	Effect of BMI described as $K_{in}\_MCH\times {\left(\frac{BMI}{26.8}\right)}^{\theta }$	−0.0583	14.9
​	Effect of black race described as factor on $K_{in}\_MCH$	0.948	0.719
Covariates K_in__RBC	Effect of male sex described as factor on $K_{in}\_RBC$	1.07	0.484
​	Effect of age described as $K_{in}\_RBC\times {\left(\frac{AGE}{59}\right)}^{\theta }$	−0.0592	10.7
​	Effect of BMI described as $K_{in}\_RBC\times {\left(\frac{BMI}{26.8}\right)}^{\theta }$	0.0451	26.5
​	Effect of asian race described as factor on $K_{in}\_RBC$	0.970	0.610
​	Effect of ALT described as $K_{in}\_RBC\times {\left(\frac{ALT}{18}\right)}^{\theta }$	0.0169	20.0
Random effects			
Hemoglobin load	IIV MCH production rate (CV%)	5.76	4.40
​	IIV AUC_50_ of inhibitory drug effect (CV%)	83.0	10.9
​	Additive residual error MCH (pg/cell)	0.522	1.44
Red blood cells	IIV RBC production rate (CV%)	7.49	2.41
​	IIV feedback on RBC production rate (CV%)	79.7	20.2
​	Additive residual error RBC (10^12^/L)	0.159	1.38
CV% $=100\times \sqrt{\left(\exp\left(\omega^{2}\right)-1\right)}$ . θ: Covariate effect of the continuous covariates, ALT: Alanine transaminase levels (liver damage biomarker), AUC_50_: Area under the drug concentration-time curve at steady state leading to half of the maximum drug effect, BMI: Body mass index, E_max_: Maximum drug effect, IIV: Interindividual variability, K_in__MCH: MCH production rate, K_in__RBC: RBC production rate, MCH: Mean corpuscular hemoglobin per cell, RBC: Red blood cell count (10^12^/L), RSE: Relative standard error.			

Red blood cell lifespan was fixed to a literature value from healthy volunteers of 126 days (Zhang et al., 2018), because Phase II clinical data were not collected beyond 84 days and therefore did not support estimation of this parameter. The applicability of this value was confirmed using the data from the Phase III trials as an external validation cohort (please see Section 3.4 “Model validation”).

All parameters were estimated with good precision (<30% RSE). Goodness-of-fit plots are shown in the Supplementary Material (Supplementary Figures S2, S3). Exemplary plots of individual predicted MCH and RBC profiles compared to clinical data are provided in Supplementary Figures S4, S5. Dose-stratified visual predictive checks (VPCs) comparing the model-predicted MCH and RBC values with the observed clinical data from the Phase II CIAS study show that the model was able to generate data similar to the observed data to which it was fit (Supplementary Figures S6, S7).

### 3.2 Covariate effects

The identified PK covariates implemented into the final popPK model are shown in Supplementary Table S2. The geometric mean AUC_τ,ss_ was 4020 nmol*h/L (64.5 %CV) for a typical CIAS patient taking 10 mg iclepertin once daily (Supplementary Table S3). Individual subject’s Bayes estimates of AUC_τ,ss_ generated with this popPK model were used as exposure input for popPD modeling. In the popPD model, covariates were implemented using a normalized power model for continuous covariates and a proportional model for categorical covariates. Distributions of the investigated continuous covariates within the model building dataset are shown in the Supplementary Material (Supplementary Figure S10).

The following covariates were identified as statistically significant and included in the model: age, BMI, and Black race on K_in__MCH, as well as sex, age, BMI, Asian race, and ALT level on K_in__RBC (see model structure in Figure 1). Implementation of smoking status and alcohol consumption as covariates on K_in__MCH also improved the model fit in a statistically significant manner (p<0.05), but the effects were too small to be meaningful compared to the observed inter- and intra-subject variability in the observed data. No statistically significant correlation with estimated glomerular filtration rate, serum creatinine, total serum protein, clinical diagnosis or dose was found. Figure 2 shows the covariate effects on hemoglobin levels after 1 year of iclepertin treatment.

### 3.3 Model-based simulations

Patient population simulations of the hemoglobin concentration-time course under chronic iclepertin treatment (365 days) at the intended therapeutic dose of 10 mg daily, as well as at a fivefold higher steady state exposure (potential extreme scenario, e.g., due to co-administration of a strong CYP3A4 inhibitor) are presented in Figure 3. Simulations predicted a new, lower hemoglobin steady state reached after approximately 120–140 days, and that more than 97.5% of the patients stayed above the drug discontinuation safety threshold of 100 g/L hemoglobin even during the high exposure scenario. This threshold was considered clinically safe for both sexes in the target patient population and equaled the threshold between mild and moderate anemia in the WHO guidance at that time (DeMaeyer et al., 1989). Nevertheless, reporting of anemia as an adverse event was always based on Principal Investigator’s decision and clinical judgement, as clinical significance of anemia can only be assessed on the individual patient level, independently from the clinical laboratory values. For both male and female reference patients (White, 38 years of age, BMI = 29.1 kg/m2, ALT = 21.0 U/L), relative hemoglobin decreases of 2.0% are predicted at 10 mg iclepertin daily and 7.6% are predicted at a fivefold higher exposure. These equate to absolute decreases of 2.9 g/L or 11.3 g/L for a male reference patient, and 2.7 g/L or 10.5 g/L for a female reference patient.

In Figures 4, 5, sex, race and age were varied simultaneously to investigate the risk of impeded hemoglobin synthesis in different patient subgroups. The simulations predicted that during chronic treatment with 10 mg iclepertin daily more than 97.5% of the virtual patients stayed above the hemoglobin safety threshold for all subgroups, even the Black, 90-year old female patients, illustrating the utility of the model for prospective simulations.

### 3.4 Model validation

**FIGURE 6:**
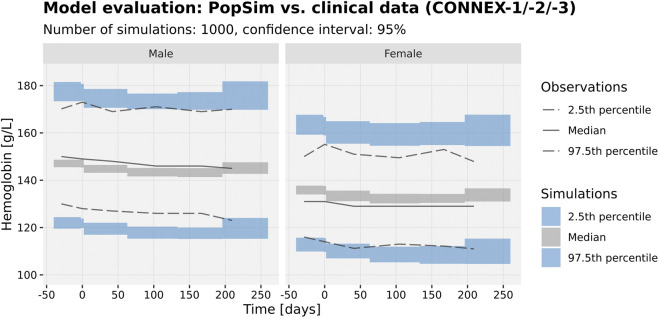
Model validation using clinical Phase III hemoglobin data from CIAS patients treated with 10 mg iclepertin daily. Blue shaded areas: 95% confidence intervals of the 2.5th and 97.5th prediction percentiles, grey shaded areas: 95% confidence intervals of the median predictions. Dashed lines: 2.5th and 97.5th percentiles of the observations, solid lines: medians of the observations.

Figure 6 shows the performance of the model developed based on Phase II data compared to the observed Phase III hemoglobin measurements. Patient hemoglobin concentrations and the trend of the data are well predicted, with a slight over-prediction of hemoglobin levels in females, demonstrating that the model generalizes well to a new sample of CIAS patients and can extrapolate to 182 days using the Phase II model parameter estimates.

## 4 Discussion

In the presented study a popPKPD model was built to describe the exposure-safety relationship of the GlyT1 inhibitor iclepertin with patient hemoglobin levels at daily doses ranging from 2 to 25 mg over a duration of up to 84 days. Subsequently, the ability of the model to predict future hemoglobin data was confirmed using the 182-day treatment Phase III CONNEX trials when these became available. This is the first model of the relationship between iclepertin exposure and hemoglobin levels, and, to our best knowledge, the first model characterizing a drug effect on hemoglobin levels over time that has been validated with clinical data that extend beyond the erythrocyte life span of ∼126 days.

The new model was applied to assess the hypothetical risk of anemia in patients and vulnerable patient subgroups during a full year of treatment. The investigation of supratherapeutic exposure and subgroups with multiple unfavorable covariates showed that the effect on hemoglobin is small (>97.5% of the virtual patients staying above the 100 g/L hemoglobin). In addition, it is reversible and can be easily monitored for patients under higher risk. However, hemoglobin restoration to pretreatment levels is as slow as its decrease when treatment is initiated (120–140 days) as it also depends on the erythrocyte turnover.

Our structural model is very similar to the one presented by Schaedeli-Stark et al. (2012). The models published by Hamrén et al. (2008) and Rognås et al. (2025) are more complex but also use two parallel linked chains of four transit compartments each to represent the red blood cells and their hemoglobin content. Comparing iclepertin to bitopertin as GlyT1 inhibitors from the same drug class, our results are very consistent with those reported for bitopertin, with similar E_max_ values for the drug effects on hemoglobin (iclepertin estimated at 0.55, bitopertin fixed at 0.6), and a slightly higher AUC_50_ for iclepertin (iclepertin estimated at 43500 nmol*h/L, bitopertin estimated at 30400 nmol*h/L) (Rognås et al., 2025).

### 4.1 Model parameter estimates

To evaluate the extent of the modeled drug impact against data from GlyT1 knock-out mice, simulations at exposures far beyond the expected therapeutic range were conducted. Defined by the estimated maximum drug effect of 0.552 (Table 2), the maximum predicted hemoglobin decrease approached ∼25%, which compares well with literature data of double GlyT1 knock-out mice that showed a 26% decrease in blood hemoglobin concentrations (Schranzhofer et al., 2011). Treatment of rats with the GlyT1 inhibitor bitopertin was reported to produce a 20% reduction in hemoglobin at steady state (Winter et al., 2016). This limited effect could be explained by glycine uptake into the erythrocyte precursors via other, lower affinity glycine transporters that are not affected by GlyT1 knock-out or selective inhibition of GlyT1.

### 4.2 Model performance

The model performance against the new Phase III hemoglobin measurements showed a slight over-prediction of hemoglobin levels in female patients. The share of female patients randomized into the CONNEX-1/-2/-3 trials was 34% of all patients (Keefe et al., 2025). The clinical data in Figure 6 illustrate that the effects of 10 mg iclepertin daily on hemoglobin levels were small and that the hemoglobin concentrations in the Phase III CIAS patients showed a lower variability compared to the Phase II dataset, which included CIAS and Alzheimer’s patients with a much broader range of ages. Overall, the model was able to describe the Phase III data well. The over-prediction of the variability observed in Figure 6 is likely driven by the less heterogenous hemoglobin levels in the Phase III data.

A limitation of the presented validation is that the model’s predictive performance was assessed exclusively against 10 mg Phase III data. Additional validation covering the whole model building dose range (2–50 mg) would be ideal. Nonetheless, the Phase III dataset applied for validation included individual patient exposures from 465–19202 nmol*h/L (typical exposure in the 10 mg dose group Phase II CIAS patients was 4020 nmol*h/L). The current evidence supports the model’s fitness for its intended purpose to inform dosing at the expected therapeutic dose of 10 mg daily in CIAS patients.

### 4.3 Model limitations

Limitations of the presented model are that we combined data from healthy individuals, CIAS patients and Alzheimer’s disease patients for model development, assuming that there was no difference in the role of GlyT1 in hemoglobin synthesis between these populations. We tested “medical diagnosis” as a covariate but could not find any evidence for differences after implementing age as the more significant covariate. Therefore, the model cannot distinguish between healthy individuals, CIAS patients or Alzheimer’s disease patients, should there be a difference. Furthermore, the red blood cell lifespan was fixed to a literature value from healthy volunteers (Zhang et al., 2018), due to lack of data collected past 84 days in the clinical studies used to build the model. This imposes the assumptions (i) that there is no difference in RBC lifespan between the healthy volunteers, CIAS patients and patients with Alzheimer’s disease included for model development, and (ii) that there is no change of RBC lifespan due to iclepertin treatment.

However, the external validation showed that the model was able to generalize well to a new sample of CIAS patients and that the model can extrapolate to 182 days accurately. Similar values for RBC lifespan were shown in the bitopertin analysis, where it was estimated at 126 days in healthy volunteers treated with bitopertin (Schaedeli-Stark et al., 2012). Other published models estimated the lifespan of red blood cells at 125 days (Rognås et al., 2025) and 135 days (Hamrén et al., 2008). Together, these findings provide evidence that the assumptions of conserved role of GlyT1 in hemoglobin synthesis and of unchanged RBC lifespan across healthy volunteers, patients and subjects treated with GlyT1 inhibitors were not invalid.

### 4.4 Covariate effects

In addition to the well-known differences between men and women regarding their hemoglobin concentrations in the blood (resulting in different standard ranges), race, age, BMI and ALT levels were found to correlate in a statistically significant manner with the hemoglobin levels. Female, Black, Asian, elderly, overweight, and patients with normal ALT levels exhibited lower hemoglobin concentrations both at baseline and under chronic iclepertin treatment. However, none of the investigated covariate effects were strong enough to raise safety concerns during chronic treatment with 10 mg iclepertin daily.

The most impactful covariates found were sex and race. The identified correlations of Black race and Asian race with hemoglobin levels (5.2% and 3.0% lower levels, respectively) are consistent with previously published hemoglobin concentrations summarized by race group (Lim et al., 2015). The effect of BMI on hemoglobin was small (1.9% difference in hemoglobin from the lowest to the highest BMI in the Phase II CIAS trial population). Higher ALT values were associated with higher hemoglobin concentrations. While erythropoiesis occurs in the bone marrow, the spleen and liver remove defective red blood cells and break down the hemoglobin to recycle the iron from the heme groups. Therefore, ALT and AST as markers of liver damage were investigated in the covariate analysis. No statistically significant correlation with kidney function could be identified, which was surprising, as erythropoietin is secreted by the kidneys in response to cellular hypoxia to stimulate red blood cell production in the bone marrow. This might signify that the decrease in hemoglobin levels during treatment with 10 mg iclepertin daily is too small to affect tissue oxygenation in the investigated patients, which is possible given the high oxygen-carrying capacity of hemoglobin.

Further identified covariates were smoking and alcohol consumption. The implementation of these extrinsic factors improved the model fit in a statistically significant manner (p<0.05). However, although it may be physiologically plausible that smoking and alcohol consumption increase hemoglobin blood concentration (smoking decreases the oxygen transport capacity of hemoglobin via carbon monoxide exposure, and alcohol consumption negatively affects liver cells) and have been reported in previous studies (Milman and Pedersen, 2008), their covariate effects (2% and 1% increase in hemoglobin, respectively) were very small compared to the large hemoglobin variability. Therefore, they were not retained in the final model.

## 5 Conclusion

A popPKPD model to describe and predict the impact of iclepertin exposure on hemoglobin concentrations was successfully developed. This model provides a generalizable modeling and simulation framework to assess the risk of anemia in patients and vulnerable patient subgroups during untested clinical scenarios and to inform the need for monitoring.

The results of this analysis suggest that iclepertin effects on patient hemoglobin levels were small, reversible, and of limited clinical significance even during a full year of treatment, in line with the results of nonclinical studies and conducted clinical trials. Clinically, changes of hematological parameters must always be interpreted for each individual patient by the treating physician based on the actual clinical condition and environment as well as local requirements and guidelines.

## Data Availability

The original contributions presented are included in the article/Supplementary Material, further inquiries can be directed to the corresponding author.
