# Supplementary material for: Pharmacokinetic/pharmacodynamic analysis to characterize the effect of long-term GlyT1 inhibitor iclepertin exposure on hemoglobin levels
Source: Front Pharmacol. 2026 Jul 10;17:1770980. doi: 10.3389/fphar.2026.1770980 (PMC13396213; doi:10.3389/fphar.2026.1770980)
Supplement: Supplementary file 1 [file DataSheet1.pdf]

Supplementary Material

Pharmacokinetic/pharmacodynamic analysis to characterize the effect of long-term GlyT1 inhibitor iclepertin exposure on hemoglobin levels

Nina Hanke[1*], Samuel P. Callisto[2], Peter Nagy[3], Michael Desch[4], Mahmoud Tareq Abdelwahab[4]

[1]Global Clinical Pharmacology & Non-clinical Safety Science, Boehringer Ingelheim Pharma GmbH & Co. KG, Ingelheim, Germany

[2]Metrum Research Group, Boston, MA, United States

[3]Global Patient Safety and Pharmacovigilance, Boehringer Ingelheim International GmbH, Ingelheim, Germany

[4]Global Clinical Pharmacology & Non-clinical Safety Science, Boehringer Ingelheim Pharma GmbH & Co. KG, Biberach, Germany

*****Corresponding Author: nina.hanke@boehringer-ingelheim.com

1 Population pharmacokinetic (popPK) model

Table S1 shows the clinical studies used for popPK model development. Parameter estimates of the final popPK model are listed in Table S2. Table S3 presents the steady state exposures for the Phase II in CIAS patient population, generated using this model.

Supplementary Table S1: Clinical data used for population PK model development

Study type	Dose groups	Treatment duration	Number of subjects (per dose group)	PK sampling
SRD	0.5, 1, 2, 5, 10, 25, 50, 100, 150 mg	SD (+ 8 days)	n = 65 (6/6/6/6/6/17/6/6/6)	0, 0.25, 0.5, 0.75, 1, 1.5, 2, 3, 4, 5, 6, 8, 10, 12, 24, 34 h, day 2, 3, 4, 5, 6, 7, 8
MRD	10, 25, 50, 75 mg daily	12 days (+ 9 days)	n = 75 (9/30/18/18)	(0, 0.5, 1, 2, 3, 3.5, 4, 4.5, 5, 6, 8, 10, 12 h)*, 24, 34, 48, 58 h, day 3-21
Proof of Mechanism	5, 10, 25, 50 mg daily	14 days (+ 3 days)	n = 25 (6/6/8/5)	(0, 0.5, 1, 2, 3, 3.5, 4, 4.5, 5, 6, 8, 10, 12 h)*, 14 h, day 1, 2, 3, 5, 7, 9, 11, 13-16
Japanese and Chinese SRD	10, 25, 50 mg	SD (+ 8 days)	n = 36 (12/12/12)	0, 0.5, 1, 2, 3, 3.5, 4, 4.5, 5, 6, 8, 10, 12, 24, 34 h, day 2, 3, 4, 5, 6, 7, 8
Itraconazole DDI	25 mg	SD (+ 8 days)	n = 16 (16)	0, 0.5, 1, 1.5, 2, 2.5, 3, 3.5, 4, 4.5, 5, 6, 7, 8, 10, 12, 24, 34 h, day 2, 3, 4, 5, 6, 7, 8
Absolute BA	25 mg	SD (+ 7 days)	n = 6 (6)	0, 1, 2, 3, 4, 6, 8, 12 h, day 1, 3, 5, 7
Human ADME	25 mg	SD (+ 48 days)	n = 6 (6)	0, 0.5, 0.75, 1, 1.5, 2, 4, 6, 8, 12, 24, 36 h, day 2-14, 20, 27, 34, 41, 48
Phase II in CIAS	2, 5, 10, 25 mg daily	84 days	n = 325 (81/79/81/84)	Pre-dose on day 21 and 42, 2x post-dose on day 42
Phase II in AD	2, 5, 10, 25 mg daily	84 days	n = 477 (120/119/119/119)	Pre-dose on day 28 and 56, 2x post-dose on day 56

Drug administration at time = 0 h @ day 0. ()*: rich sampling after first and last dose, AD: Alzheimer's disease, ADME: absorption, distribution, metabolism, excretion, BA: bioavailability, CIAS: cognitive impairment associated with schizophrenia, DDI: drug-drug interaction, MRD: multiple rising doses, SD: single dose, SRD: single rising dose. Placebo groups excluded for model development. National Clinical Trial numbers: NCT02068690, NCT02337283, NCT02362516, NCT02383888, NCT02342717, NCT03783000, NCT03654170, NCT02832037, NCT02788513.

Supplementary Table S2: Parameter estimates of the final population PK model

		Parameter (unit)	Estimate	95% CDI
Fixed effects				
	CL/F	Apparent clearance (L/h)	3.98	(3.70, 4.28)
	V2/F	Apparent central compartment volume (L)	120	(109, 133)
	Q/F	Apparent intercompartmental clearance (L/h)	6.99	(5.91, 8.14)
	V3/F	Apparent peripheral compartment volume (L)	86.2	(77.5, 95.3)
Weight effects	CL_{WT}	Effect of body weight on CL/F	0.164	(0.0120, 0.321)
	$V2_{WT}$	Effect of body weight on V2/F	0.864	(0.673, 1.05)
	Q_{WT}	Effect of body weight on Q/F	0.750	Fixed
	$V3_{WT}$	Effect of body weight on V3/F	1.00	Fixed
Absorption	KA	Absorption rate constant (1/h)	0.383	(0.318, 0.462)
	ALAG	Lag time into depot (h)	0.233	(0.230, 0.235)
Food effects	KA_{FED}	Effect of food on KA	1.68	(1.48, 1.90)
	F_{FED}	Effect of food on BA	1.15	(1.10, 1.20)
	F1-10mg	Relative BA for 10 mg doses	0.931	(0.872, 0.998)
	F1-25mg	Relative BA for 25 mg doses	0.823	(0.775, 0.873)
Covariates	CL_{OTHER}	CL for subjects of other race	0.963	(0.793, 1.17)
	CL_{ASIAN}	CL for subjects of Asian race	1.00	(0.910, 1.10)
	CL_{BLACK}	CL for subjects of Black race	1.35	(1.19, 1.53)
	CL_{AGE}	Effect of age on CL	-1.64	(-2.07, -1.21)
	CL_{SMOKE}	Effect of smoking on CL	1.02	(0.935, 1.12)
	KA_{DIAG}	Effect of diagnosis on KA	2.31	(1.69, 3.29)
Random effects				
IIV CL/F	ω^2 CL/F	Interindividual variability of CL/F	0.242 (52.4%CV)	(0.218, 0.270)
	COV_{CL-V2}	Covariance of CL/F and V2/F	-0.0371 (-0.220)	(-0.0744, -0.00506)
	COV_{CL-KA}	Covariance of CL/F and KA	-0.171 (-0.343)	(-0.264, -0.104)
IIV V2/F	ω2 V2/F	Interindividual variability of V2/F	0.118 (35.4%CV)	(0.0839, 0.179)
	COV_{V2-KA}	Covariance of V2/F and KA	0.227 (0.657)	(0.135, 0.416)
IIV KA	ω^2 KA	Interindividual variability of KA	1.03 (134%CV)	(0.753, 1.71)
Residual error	σ^2 PROP	Proportional residual error	0.0919 (30.3%CV)	(0.0884, 0.0956)

BA: bioavailability, CDI: Bayesian estimation credible intervals, COV: covariance (correlation), IIV: interindividual variability.

Supplementary Table S3: Steady state exposure predictions for the Phase II in CIAS patient population generated with the final population PK model

Dose	$AUC_{\tau,ss}$ [nmol*h/L]	$C_{max,ss}$ [nmol/L]	$C_{trough,ss}$ [nmol/L]
2 mg	929 (47.1%)	50.1 (37.4%)	29.2 (61.6%)
5 mg	2090 (58.0%)	113 (46.6%)	65.1 (75.1%)
10 mg	4020 (64.5%)	222 (43.9%)	121 (104%)
25 mg	8390 (44.1%)	462 (32.8%)	255 (62.7%)

$AUC_{\tau,ss}$: area under the concentration-time curve at steady state, $C_{max,ss}$: maximum concentration at steady state, $C_{trough,ss}$: trough concentration at steady state. Data are summarized as geometric means (with geometric CV).

2 Clinical data used for popPD model development

Data from three different clinical trials were used to build the popPD model and to analyze the covariate correlation. The model was fitted to individual mean corpuscular hemoglobin measurements (MCH) and red blood cell counts (RBC). For clinical relevance the individual measured hemoglobin concentrations (HGB=MCH*RBC) are shown in Figure S1.

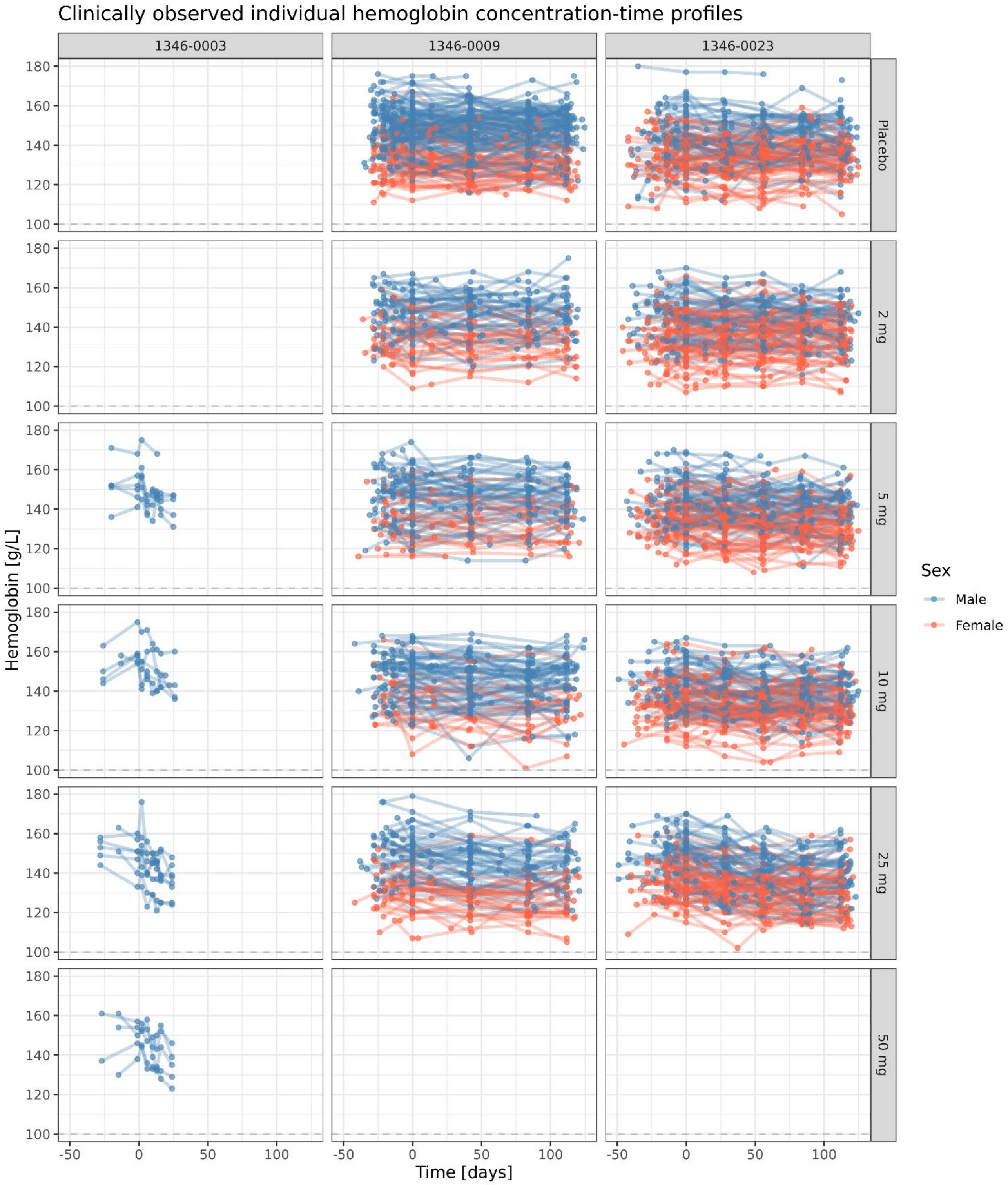

Supplementary Figure S1: Individual observed hemoglobin-time profiles used to develop the popPD model and analyze the covariates. Dashed lines indicate the drug discontinuation safety threshold of 100 g/L hemoglobin.

Table S4 shows the number of hemoglobin (HGB) measurements per sampling time from the global CONNEX Phase III trials utilized for model validation (placebo patients excluded), with fewer available samples from female patients.

Supplementary Table S4: Number of measurements per sampling time used for model validation

Phase III CONNEX-1/-2/-3	Baseline	Week 6	Week 12	Week 26	Follow-up
Male	544	556	486	447	420
Female	277	276	259	229	211

26 weeks treatment with 10 mg iclepertin daily. Placebo groups excluded. The number of available measurements for HGB, RBC and MCH is identical. CONNEX-1: NCT04846868, CONNEX-2: NCT04846881, CONNEX-3: NCT04860830.

3 Run record

In the final model, GlyT1 inhibition by iclepertin was implemented as proportional drug effect on the MCH production rate, using an E_{max} model. The objective function values of the different tested drug effect models are shown in Table S5.

Supplementary Table S5: Run record comparing the objective function values of the different tested drug effect models to the final model as reference

Description	OFV	ΔOFV	Sig.Digits	Notes
**Proportional drug effect, E_{max} inhibition model**	**-9272.82**	**-**	**4.0**	**Final model**
Proportional drug effect, linear inhibition model	-9246.98	+25.8	3.0	Δ vs. final
Subtractive drug effect, E_{max} inhibition model	-9253.52	+19.3	3.1	Δ vs. final
Subtractive drug effect, linear inhibition model	-9230.80	+42.0	3.1	Δ vs. final

OFV: objective function value, ΔOFV: difference in objective function value compared to final model, Sig.Digits: number of precise decimal places achieved for the "least-well-determined" parameter estimate. Minimization and covariance steps were successful for all listed runs.

4 NONMEM control stream

```
$SIZES LIM1=40000 LIM3=2000 LIM4=2000 PAL=200 PD=-1000 LVR=-150 LTH=-200

$PROB Iclepertin drug effect on hemoglobin

$INPUT C ID

$DATA pk-safety-pd.csv IGNORE=@

$SUBROUTINE ADVAN6 TOL=7

$MODEL
COMP=(MCH1) ;1 Mean corpuscular Hb compartment 1
COMP=(MCH2) ;2 Mean corpuscular Hb compartment 2
COMP=(MCH3) ;3 Mean corpuscular Hb compartment 3
COMP=(MCH4) ;4 Mean corpuscular Hb compartment 4
COMP=(RBC1) ;5 Red blood cell compartment 1
COMP=(RBC2) ;6 Red blood cell compartment 2
COMP=(RBC3) ;7 Red blood cell compartment 3
COMP=(RBC4) ;8 Red blood cell compartment 4

$PK
STUDY_FLAG= STUDY*1000+FLAG

TVLS = THETA(1) ;Lifespan RBC (days)
TVKinMCH = THETA(2) ;Hemoglobin (Hb) production rate per RBC (pg/cell/day)
TVKinRBC = THETA(3) ;Red blood cell (RBC) production rate (10^12/L/day)
TVFeedbk = THETA(4) ;Stimulation of RBC production rate as feedback to Hb decrease ( )
TVEmax = THETA(5) ;Emax of inhibitory drug effect ( )
TVAUC50 = THETA(6) ;AUC50 of inhibitory drug effect (nmol*h/L)

SexEff = THETA(7) ;Effect of male sex ( )
RACEBL = 0 ;All ethnicities except for black
IF (RACE.EQ.1) RACEBL= 1 ;When subject is black (RACE=1) set new variable RACEBL to 1
RaceEff = THETA(10) ;Effect of black ethnicity
AgeEff = THETA(11) ;AGE on KinRBC_0
BMIEff = THETA(12) ;BMI on KinMCH
BMIEff2 = THETA(13) ;BMI on KinRBC_0
RACEASIA = 0 ;All ethnicities except for Asian
IF (RACE.EQ.2) RACEASIA=1 ;When subject is Asian (RACE=2) set new variable RACEASIA to 1
RaceEff2 = THETA(14) ;RACE2 on KinRBC_0
AgeEff2 = THETA(15) ;AGE2 on KinMCH
ALTEff = THETA(16) ;ALT on KinRBC_0

ELS = ETA(1) ;Lifespan RBC (days)
EKinMCH = ETA(2) ;Hemoglobin (Hb) production rate per RBC (pg/cell/day)
EKinRBC = ETA(3) ;Red blood cell (RBC) production rate (10^12/L/day)
EFeedbk = ETA(4) ;Stimulation of RBC production rate as feedback to Hb decrease ( )
EEmax = ETA(5) ;Emax of inhibitory drug effect ( )
EAUC50 = ETA(6) ;AUC50 of inhibitory drug effect (nmol*h/L)

LS = TVLS*EXP(ELS)
KinMCH = TVKinMCH*EXP(EKinMCH) *(RaceEff**RACEBL) *((BMI/26.8)**BMIEff)*((AGE/59)**AgeEff2)
KinRBC_0 = TVKinRBC*EXP(EKinRBC) *(SexEff**(1-SEX)) *((AGE/59)**AgeEff)*((BMI/26.8)**BMIEff2)
*(RaceEff2**RACEASIA) *((ALT/18)**ALTEff)
```

```
Feedbk = TVFeedbk*EXP(EFeedbk)
Emax = TVEmax*EXP(EEmax)
AUC50 = TVAUC50*EXP(EAUC50)

;-------------- Transit rate and compartment initialization ----------
Ktr = 4/LS;
A_0(1) = KinMCH/Ktr;
A_0(2) = KinMCH/Ktr;
A_0(3) = KinMCH/Ktr;
A_0(4) = KinMCH/Ktr;
A_0(5) = KinRBC_0/Ktr;
A_0(6) = KinRBC_0/Ktr;
A_0(7) = KinRBC_0/Ktr;
A_0(8) = KinRBC_0/Ktr;
HB_0 = 4*(KinMCH/Ktr)*(KinRBC_0/Ktr) ;baseline hemoglobin from MCH and RBC

$DES
HB = A(1)*A(5) + A(2)*A(6) + A(3)*A(7) + A(4)*A(8) ;hemoglobin time-varying value
KinRBC = KinRBC_0*(HB/HB_0)**Feedbk
INH = (Emax*AUCSS)/(AUC50+AUCSS)

;------------- MCH compartments --------------------------------------
DADT(1) = KinMCH *(1-INH) - Ktr*A(1) ;
DADT(2) = Ktr*A(1) - Ktr*A(2) ;
DADT(3) = Ktr*A(2) - Ktr*A(3) ;
DADT(4) = Ktr*A(3) - Ktr*A(4) ;

;------------- RBC compartments --------------------------------------
DADT(5) = KinRBC - Ktr*A(5) ;
DADT(6) = Ktr*A(5) - Ktr*A(6) ;
DADT(7) = Ktr*A(6) - Ktr*A(7) ;
DADT(8) = Ktr*A(7) - Ktr*A(8) ;

AA1 = A(1)
AA2 = A(2)
AA3 = A(3)
AA4 = A(4)
AA5 = A(5)
AA6 = A(6)
AA7 = A(7)
AA8 = A(8)
MEAN = AA1 + AA2 + AA3 + AA4 ; all MCH
SUM = AA5 + AA6 + AA7 + AA8 ; all RBC

;--------MCH---------------------------------
;IPRED = LOG(1E-12)
IF(FLAG.EQ.6) IPRED = MEAN
IF(FLAG.EQ.6) W = THETA(8)

;--------RBC--------------------------------
IF(FLAG.EQ.11) IPRED = SUM
IF(FLAG.EQ.11) W = THETA(9)

;---------------------------------------------
;IF (W.LE.0.000001) W = 0.000001
IRES = DV-IPRED
IWRES = IRES/W
```

```
Y = IPRED + W*EPS(1)

;---------------------------------------------------------------------------
$THETA 126 FIX ;1: Lifespan RBC (days)
$THETA (1E-12, 0.24) ;2: Kin_MCH = MCH production rate (pg/cell/day)
$THETA (1E-12, 0.035) ;3: Kin_RBC = RBC production rate (10^12/L/day)
$THETA -1.4 ;4: Feedback on RBC production rate ( )
$THETA (1E-12, 0.55) ;5: Emax of inhibitory drug effect ( )
$THETA (1E-12, 10000) ;6: AUC50 of inhibitory drug effect (nmol*h/L)
$THETA (1E-12, 1.07) ;7: SEX on KinRBC
$THETA (1E-12, 0.52) ;8: Additive residual error MCH (pg/cell)^2
$THETA (1E-12, 0.16) ;9: Additive residual error RBC (10^12/L)^2
$THETA (1E-12, 0.95) ;10: BLACK on KinMCH
$THETA -0.06 ;11: AGE on KinRBC
$THETA -0.06 ;12: BMI on KinMCH
$THETA 0.04 ;13: BMI on KinRBC
$THETA (1E-12, 0.97) ;14: ASIAN on KinRBC
$THETA 0.03 ;15: AGE on KinMCH
$THETA 0.02 ;16: ALT on KinRBC

;---------------------------------------------------------------------------
$OMEGA BLOCK(1) FIX
0 ;IIV Lifespan
$OMEGA BLOCK(1)
0.06 ;IIV KinMCH
$OMEGA BLOCK(1)
0.08 ;IIV KinRBC
$OMEGA BLOCK(1)
0.06 ;IIV Feedback
$OMEGA BLOCK(1) FIX
0 ;IIV Emax
$OMEGA BLOCK(1)
0.7 ;IIV AUC50

;---------------------------------------------------------------------------
$SIGMA 1 FIX ;Additive errors -> THETA8, THETA9
;$SIGMA 0.522 ;Additive error on MCH (pg/cell)^2 (SD of 0.722)
;$SIGMA 0.159 ;Additive error on RBC (10^12/L)^2 (SD of 0.399)

;---------------------------------------------------------------------------
$ESTIMATION MAXEVAL=9999 PRINT=10 NOABORT METHOD=1 INTERACTION NSIG=3 SIGL=9
MSFO=../987_pretty.MSF
$COVARIANCE PRINT=E UNCONDITIONAL
```

5 Goodness-of-fit plots

Goodness-of-fit plots demonstrated a good distribution of observed versus predicted values around the line of unity for both MCH and RBC. No trend was seen in the conditional weighted residuals (CWRES), neither against predicted values nor over time (Figures S2 and S3).

For CWRES values >|4| the safety lab results of the respective patient were checked, and patients that had most (or all) of their blood parameter values (for hemoglobin, MCH, or RBC) outside their standard range (according to Lim et al., 2015) were flagged in the dataset and not considered during parameter re-estimation. In addition, patients that showed high CWRES values were excluded if their corresponding blood marker consistently showed the opposite trend than expected during the study, despite of being on active treatment. Patients that did not fulfill the above criteria were kept in the dataset, even if their observed data produced CWRES values >|4|. Applying these criteria led to the exclusion of 10 patients from the dataset, leaving the clinical data of 1116 individuals. These exclusions resulted in a model with improved OFV (reduced by > 450 units) and decreased random effects, only marginally impacting some of the parameter estimates. The distribution of the excluded patients and observations across dose groups are shown in Table S6.

Supplementary Table S6: Number of patients and observations excluded due to CWRES >|4| and concurrent blood parameter values outside their respective standard ranges

Dose	Placebo	2 mg	5 mg	10 mg	25 mg	Total
Patients excluded	1	2	5	1	1	10
Observations excluded	14	20	58	14	10	116

Final dataset for popPD model development after this exclusion: 1116 individuals, 12328 observations.

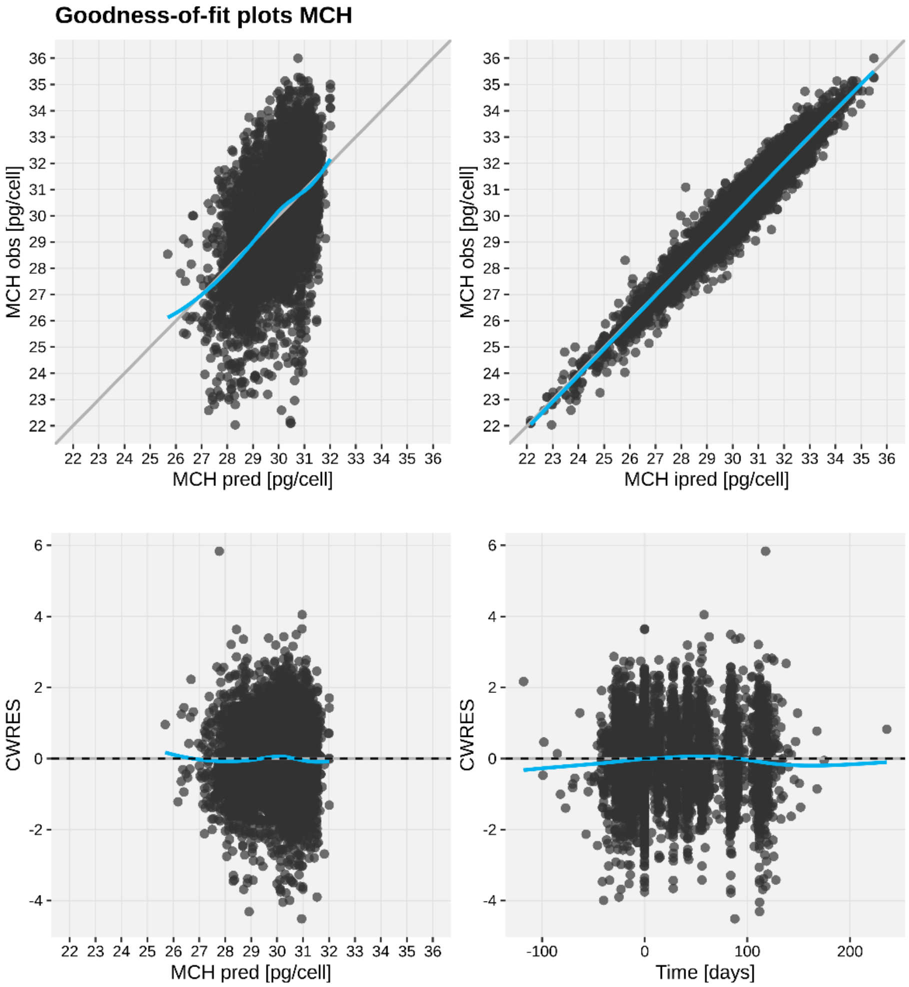

Supplementary Figure S2: Goodness-of-fit plots for mean corpuscular hemoglobin (MCH). Upper panel: (A) Observations versus population predictions or (B) versus individual predictions. Lower panel: (C) Conditional weighted residuals versus population predictions or (D) over time. Blue lines are locally estimated scatterplot smoothers (LOESS), grey lines show the line of unity, black dashed lines indicate zero.

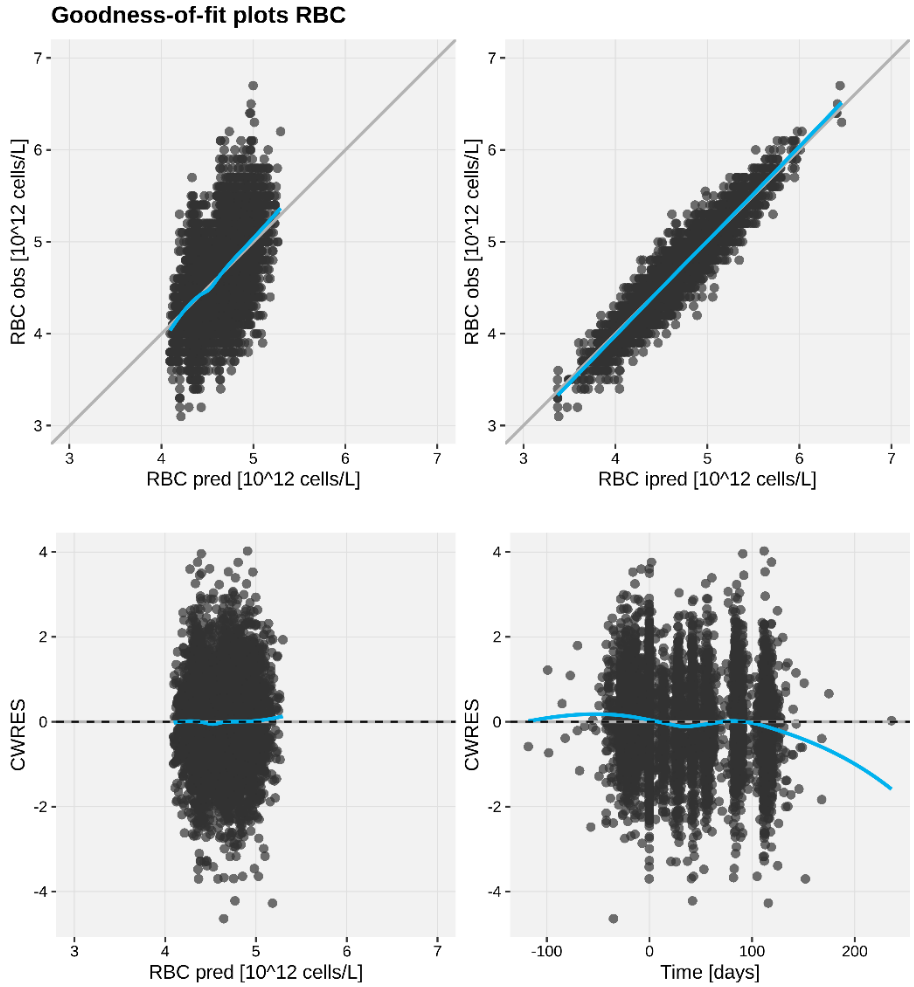

Supplementary Figure S3: Goodness-of-fit plots for red blood cell count (RBC). Upper panel: (A) Observations versus population predictions or (B) versus individual predictions. Lower panel: (C) Conditional weighted residuals versus population predictions or (D) over time. Blue lines are locally estimated scatterplot smoothers (LOESS), grey lines show the line of unity, black dashed lines indicate zero.

6 Individual plots

Exemplary plots showing observed clinical MCH and RBC data of individual patients in the highest dose group of the Phase II in CIAS trial (25 mg iclepertin daily), overlaid with the population predictions as well as the individual predictions, are shown in Figures S4 and S5.

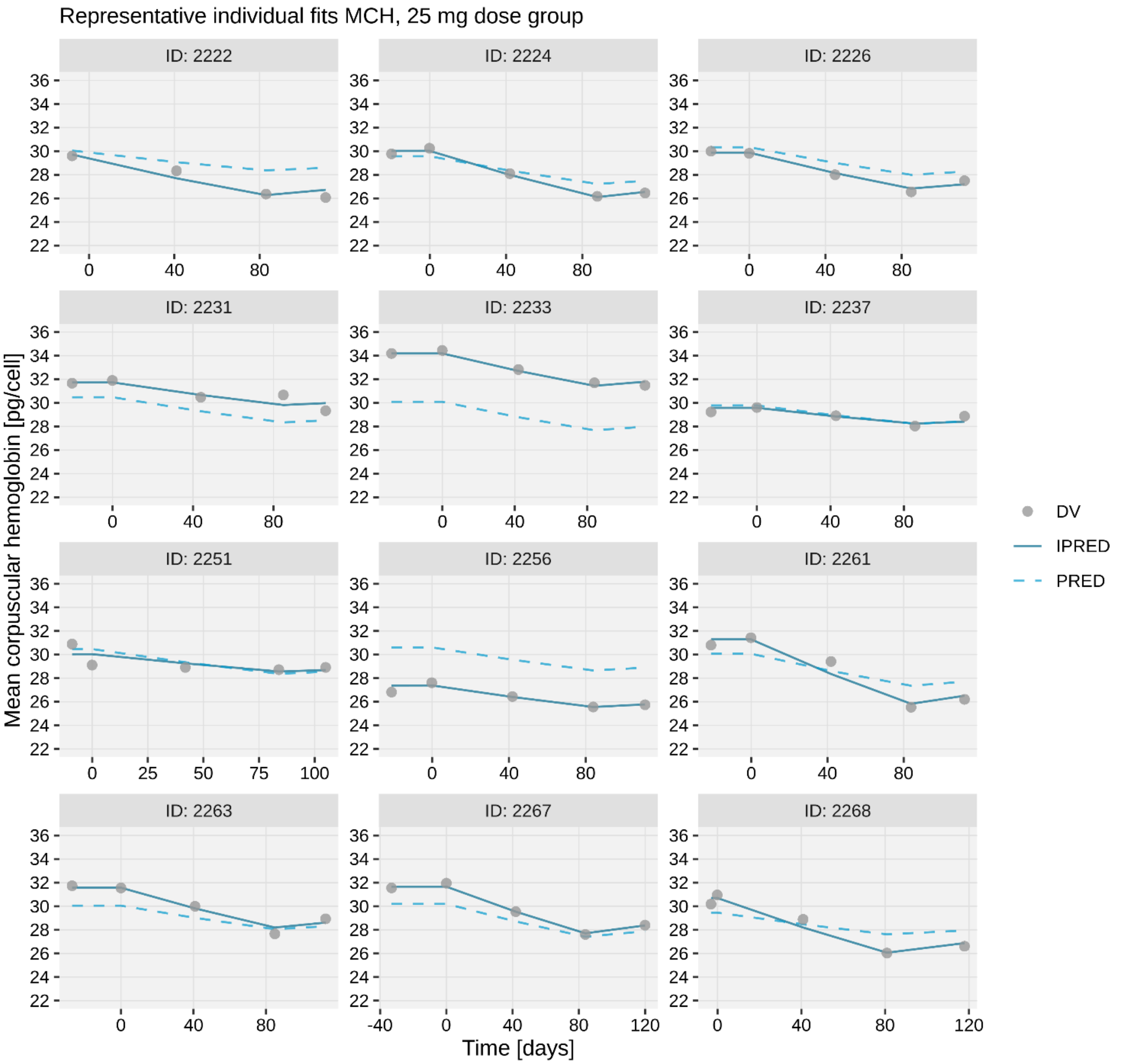

Supplementary Figure S4: Individual measured MCH values of the Phase II in CIAS trial (DV, grey dots), individual predictions (IPRED, solid blue lines) and population predictions (PRED, dashed blue lines) versus time (representative subjects from the 25 mg dose group).

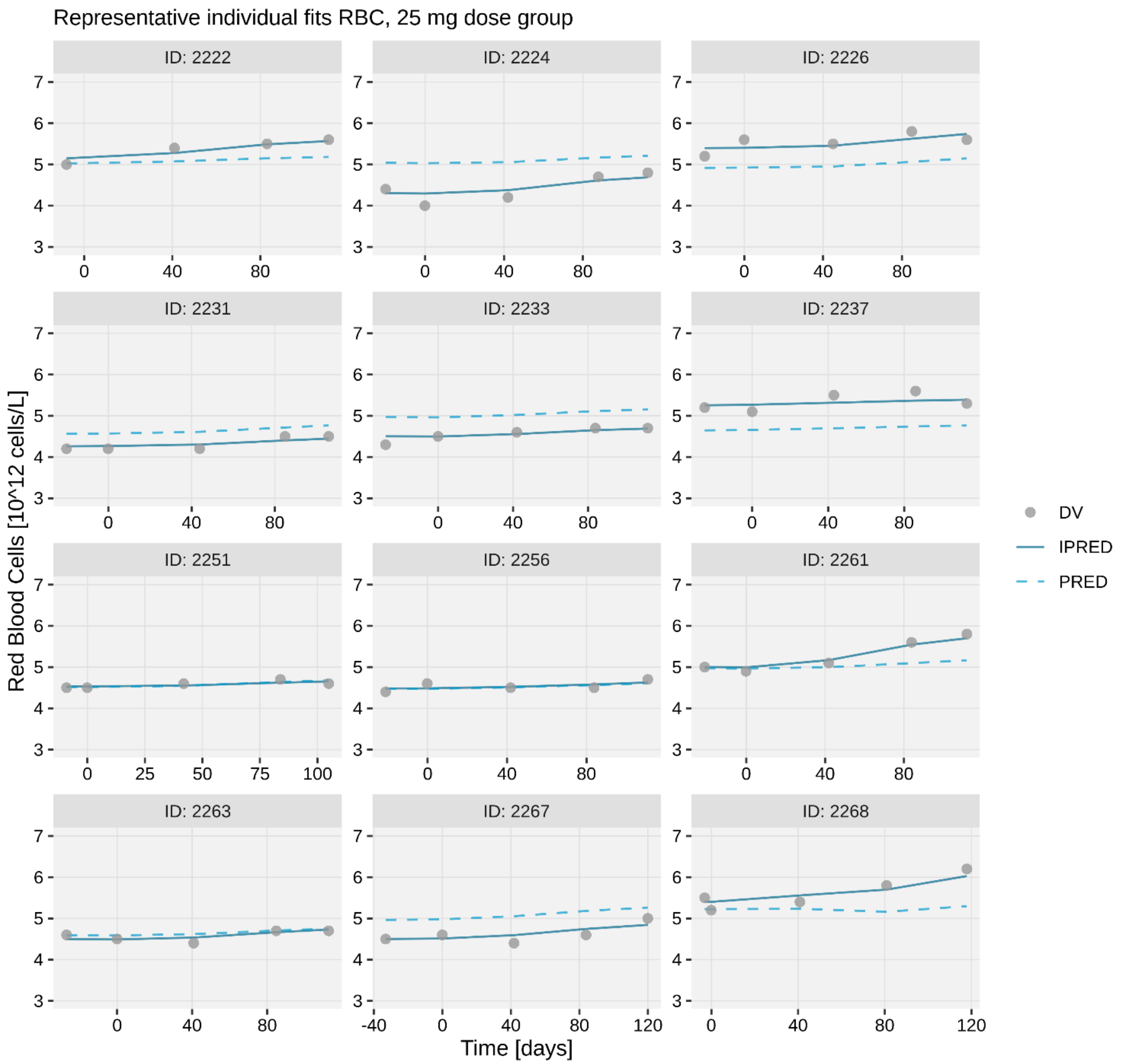

Supplementary Figure S5: Individual measured RBC values of the Phase II in CIAS trial (DV, grey dots), individual predictions (IPRED, solid blue lines) and population predictions (PRED, dashed blue lines) versus time (representative subjects from the 25 mg dose group).

7 Visual predictive checks

Dose-stratified visual predictive checks (VPCs) of the Phase II in CIAS trial comparing the model-predicted MCH and RBC values with the corresponding observed clinical data are shown below (Figures S6 and S7).

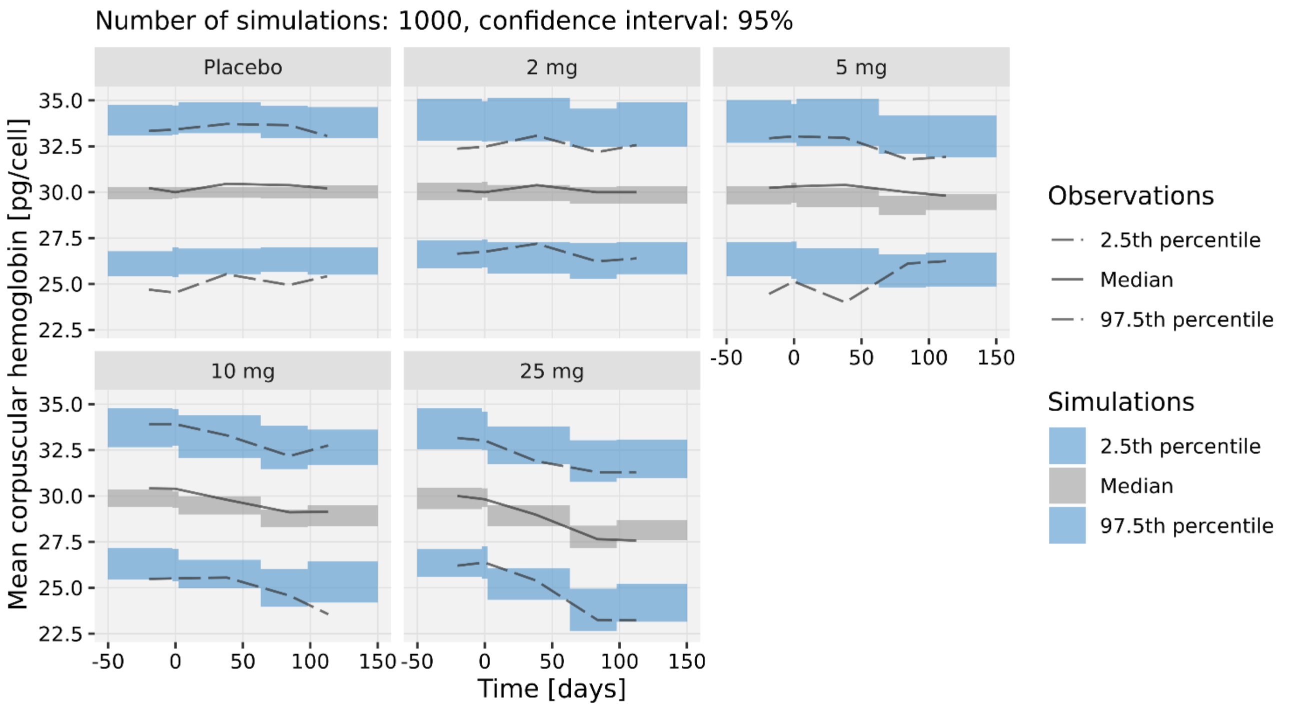

Supplementary Figure S6: Visual predictive checks of mean corpuscular hemoglobin (MCH) levels in the Phase II in CIAS trial, stratified by iclepertin dose group (Placebo: n=170, 2 mg: n=78, 5 mg: n=81, 10 mg: n=83, 25 mg: n=80). Blue shaded areas: 95% confidence intervals of the 2.5th and 97.5th prediction percentiles, grey shaded areas: 95% confidence intervals of the median predictions. Dashed lines: 2.5th and 97.5th percentiles of the observations, solid lines: medians of the observations.

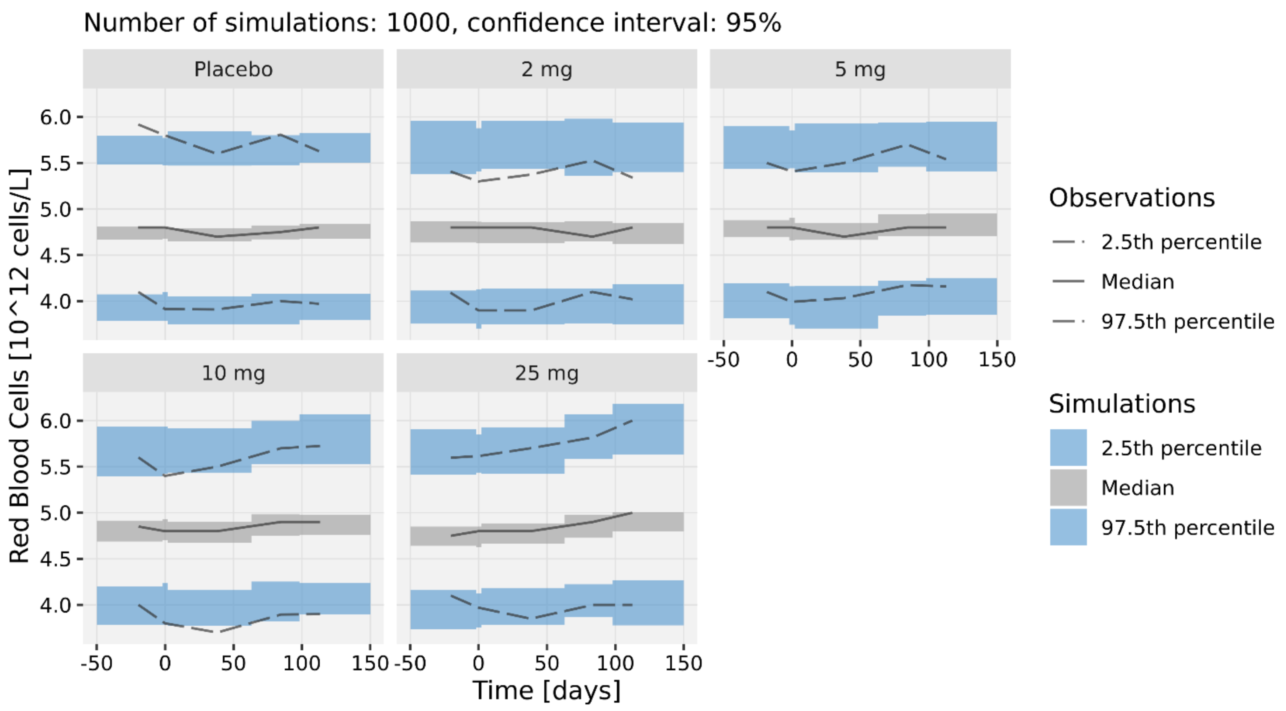

Supplementary Figure S7: Visual predictive checks of red blood cell (RBC) levels in the Phase II in CIAS trial, stratified by iclepertin dose group (Placebo: n=170, 2 mg: n=78, 5 mg: n=81, 10 mg: n=83, 25 mg: n=80). Blue shaded areas: 95% confidence intervals of the 2.5th and 97.5th prediction percentiles, grey shaded areas: 95% confidence intervals of the median predictions. Dashed lines: 2.5th and 97.5th percentiles of the observations, solid lines: medians of the observations.

8 Distributions of the residuals, parameters, random effects and continuous covariates

Conditional weighted residuals (CWRES) were centered at zero and normally distributed (Figure S8). Posterior distributions of the individual model parameters and random effects are shown in Figure S9. Distributions of the investigated continuous covariates within the model building dataset are shown in Figure S10.

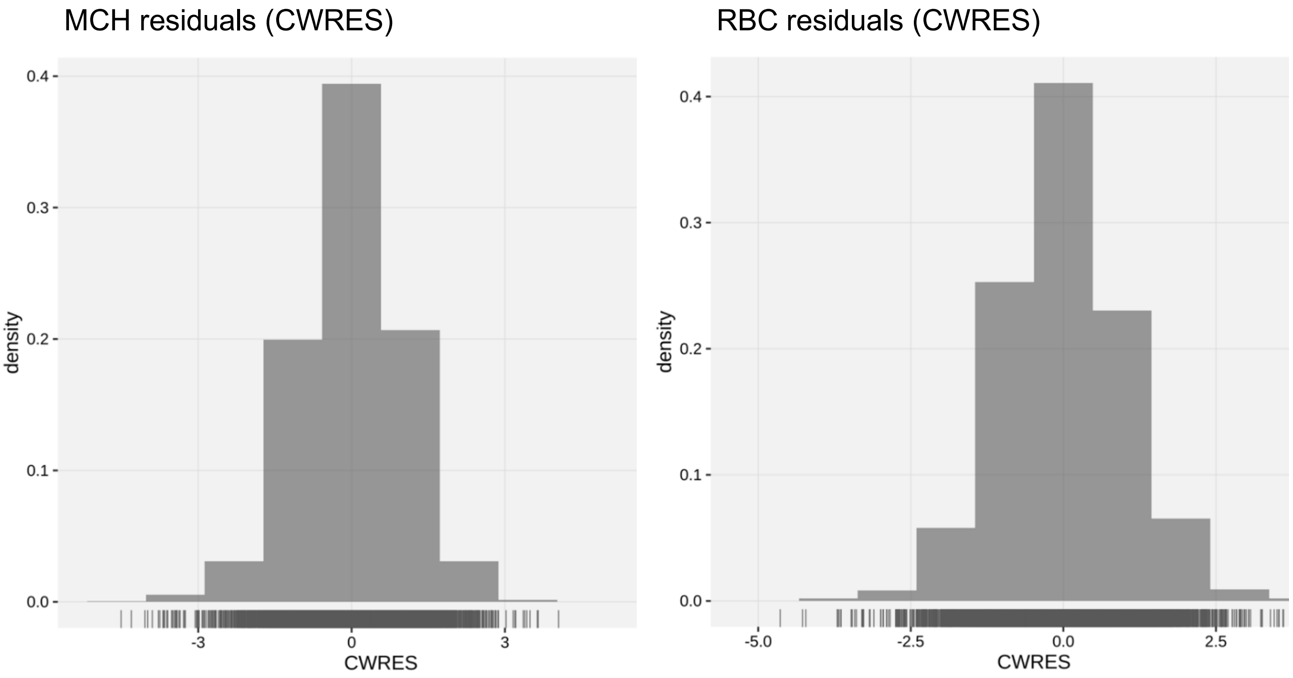

Supplementary Figure S8: Posterior distributions of the MCH residuals (left) and RBC residuals (right). CWRES: conditional weighted residuals, MCH: mean corpuscular hemoglobin, RBC: red blood cell count.

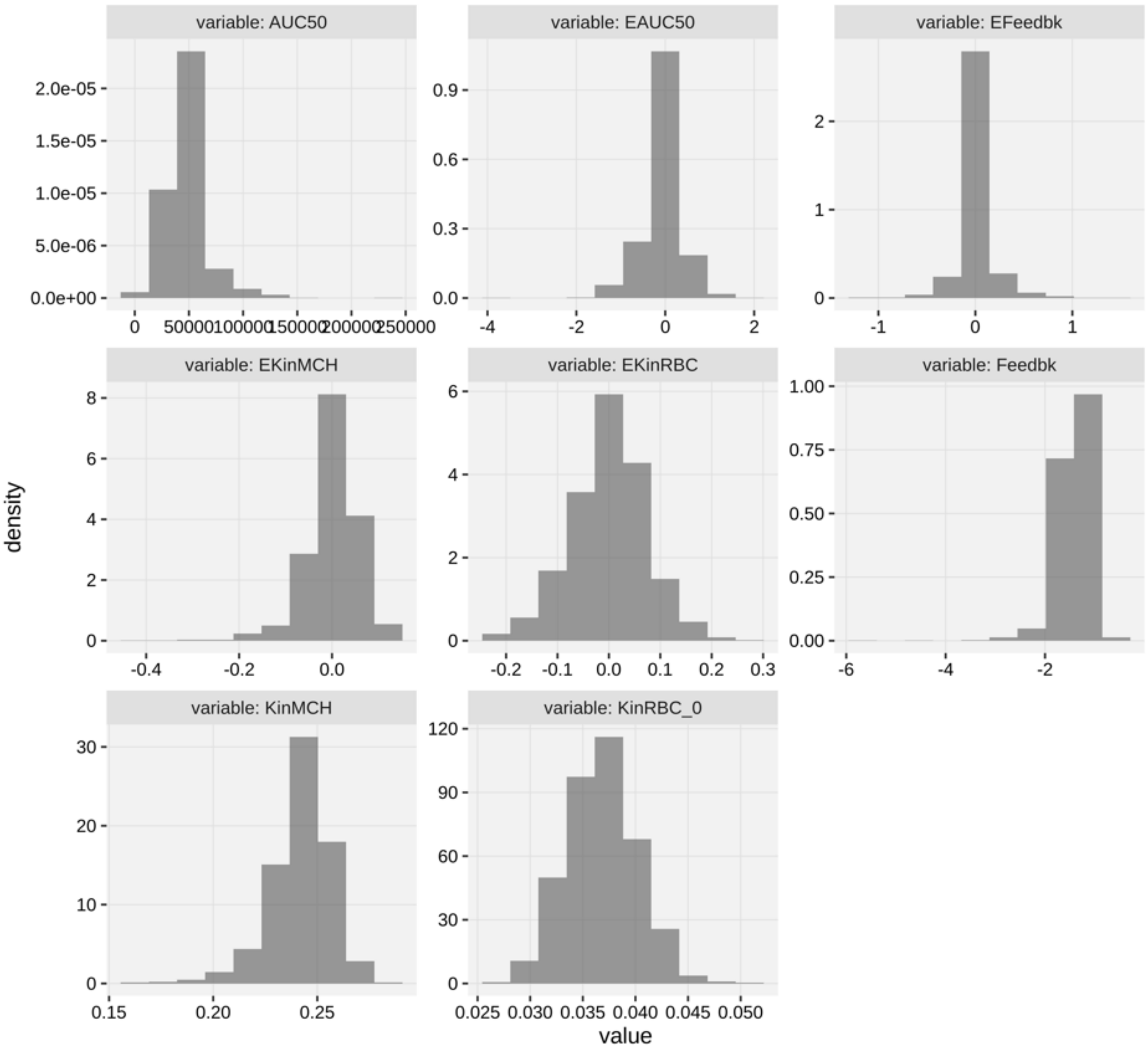

Supplementary Figure S9: Posterior distributions of the individual model parameters and random effects. AUC50: area under the drug concentration-time curve at steady state leading to half of the maximum drug effect, EAUC50: IIV AUC_{50} of drug effect, EFeedbk: IIV Feedback on RBC production rate, EKinMCH: IIV MCH production rate, EKinRBC: IIV RBC production rate, Feedbk: Feedback on RBC production rate, KinMCH: MCH production rate, KinRBC_0: baseline RBC production rate (before impact of hemoglobin feedback).

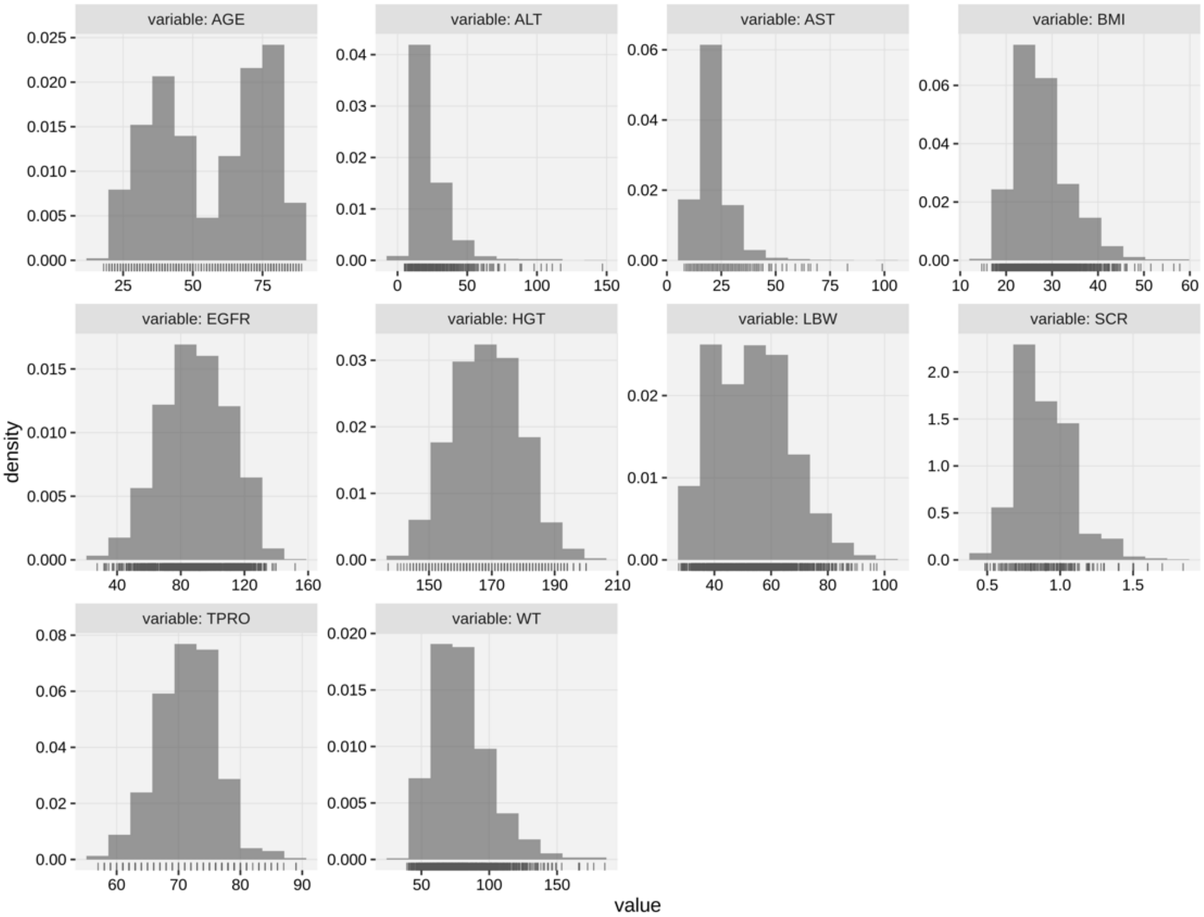

Supplementary Figure S10: Distributions of the investigated continuous covariates within the model building dataset. AGE: age (the patients in the Alzheimer's disease Phase II trial were older), ALT: alanine transaminase, AST: aspartate transaminase, BMI: body mass index, EGFR: estimated glomerular filtration rate, HGT: body height, LBW: lean body weight, SCR: serum creatinine, TPRO: total serum protein, WT: body weight.
